# Risk Factors for Cervical Lymph Node Metastasis of Papillary Thyroid Microcarcinoma: A Single-Center Retrospective Study

**DOI:** 10.1155/2019/8579828

**Published:** 2019-01-15

**Authors:** Feng Cheng, Yanyan Chen, Lei Zhu, Bin Zhou, Yonghong Xu, Yiran Chen, Liping Wen, Shuzheng Chen

**Affiliations:** ^1^Department of Thyroid and Breast Surgery, Lishui Hospital of Zhejiang University, Lishui Municipal Central Hospital, Lishui 323000, China; ^2^Department of Surgical Oncology, First Affiliated Hospital, School of Medicine, Zhejiang University, Hangzhou 310003, China

## Abstract

**Objective:**

To identify the clinicopathological features correlated to lymph node metastasis (LNM) in patients with papillary thyroid microcarcinoma (PTMC).

**Methods:**

Clinical data of 785 PTMC patients who underwent surgical treatment at the Lishui Municipal Central Hospital from September 2008 to December 2017 were retrospectively analyzed. Clinical and pathological risk factors for lymph node metastasis (LNM), central lymph node metastasis (CLNM), and lateral lymph node metastasis (LLNM) were analyzed.

**Results:**

LNM was found in 236 (30.2%) patients. Multivariate logistic regression analysis revealed that in PTMC, male gender, age < 55 years, tumor size > 5 mm, bilateral lesions, and extrathyroidal extension were independent risk factors for LNM in general and for CLNM. For LLNM, tumor size > 5 mm, multifocal lesions, and extrathyroidal extension were independent risk factors.

**Conclusions:**

Identification of risk factors for cervical LNM could assist individualization of clinical management for PTMC.

## 1. Introduction

Papillary thyroid microcarcinoma (PTMC) is defined as papillary thyroid carcinoma that is 10 mm or less in maximal diameter. Though patients with PTMC have an excellent 10-year prognosis with a survival rate of more than 99% [1], lymph node metastasis was shown to be associated with increased risk of recurrence [1–4]. The recurrence of the disease is majorly involved in lymph nodes, which may lead to additional surgery or radioiodine ablation therapy, affecting life quality of patients. Therefore, identification of patients with higher risk of LNM is necessary to optimize operation selection and subsequent therapy for PTMC.

Therapeutic or prophylactic central lymph node dissection (CLND) was recommended for treatment of PTMC when metastatic lymph nodes are evident in preoperative evaluation [5]. However, there is controversy about the necessity of routine prophylactic CLND for clinically node-negative patients [6, 7].

This study is a retrospective analysis that focuses on PTMC, aiming to analyze specific clinicopathological features that correlate with cervical lymph node metastasis (LNM) in PTMC and to provide new evidence for risk evaluation of PTMC as a guide for clinical management and subsequent follow-up.

## 2. Materials and Methods

### 2.1. Patients

The study subjects were patients who received surgical treatment at the Department of Thyroid and Breast Surgery, Lishui Municipal Central Hospital, China, from September 2008 to December 2017. The patients were included according to the following criteria: (1) no previous thyroid surgery, (2) availability of an adequate medical history, (3) underwent unilateral or bilateral central lymph node dissection, with or without lateral lymph node dissection, and (4) diagnosis of PTMC pathologically confirmed. Patients with PTMC incidentally found in thyroidectomy for benign conditions (without neck dissection) were excluded. Finally, a total of 785 patients were included in this study.

This research was approved by Medical Ethics Committee of Lishui Municipal Central Hospital, Lishui, China.

### 2.2. Surgical Treatment

Preoperative clinical and ultrasound (US) evaluations were performed for all patients. Features of malignant nodules included marked hypoechogenicity, taller-than-wide shape, spiculated margin, hypervascularity, and calcification [8]. 264 patients were diagnosed or suspected to have PTMC by fine needle aspiration (FNA). Diagnosed or suspicious malignancies were treated by surgical resection, and the final procedure was determined based on the results of frozen section examination.

The patients underwent open (624, 79.5%) or laparoscopic (161, 20.5%) thyroidectomy or lobectomy. For solitary primary lesions, lobectomy (ipsilateral lobe and isthmus resection) plus ipsilateral central lymph node dissection (CLND) was performed. For unilateral lesions which require postoperative iodine 131 treatment or bilateral lesions, total thyroidectomy plus bilateral CLND was performed. Lateral lymph node dissection (LLND) including levels II, III, IV, and Vb was performed when lymph node metastasis in lateral compartment was diagnosed or suspected by either preoperative FNA or CT. Level I and Va lymph nodes were only dissected when metastasis was present in these compartments. All surgical specimens were independently examined by two expert pathologists. Specifically, the histological type, location and number of lesions, largest diameter, and the presence of extrathyroidal extension and the number of metastatic/dissected lymph nodes were reported.

Patients received TSH suppression using levothyroxine after the surgery. Patients with any of the following criteria received iodine 131 treatment: extrathyroidal extension, metastatic lymph nodes of more than five in number or diameter > 1 cm, and postoperative unstimulated Tg level > 5 ng/mL.

### 2.3. Statistical Analysis

Statistical analysis was performed using SPSS version 20.0 (IBM, USA). Quantitative data are presented as mean ± standard deviation (SD). Chi-squared test was used to assess the difference between groups. The odds ratio (OR) and 95% confidence interval (CI) for relationships between each variable and lymph node metastasis were calculated using binary logistic regression, in which backward elimination (conditional) was used as a variable selection method. Forest plots were drawn by R software to visualize multivariate analysis results. *P* < 0.05 was considered statistically significant.

## 3. Results

### 3.1. Patient Characteristics

The baseline patient characteristics are summarized in Table 1. Among 785 patients with PTMC, 24% were male and 76% were female; the male/female ratio was 1 : 3.16. The mean age of all patients was 45.5 ± 10.0 years. The mean tumor size, defined as the maximal diameter, of this cohort was 5.73 ± 2.39 mm, while 417 (53%) patients had 5 mm PTMC or less in diameter. Based on preoperative evaluation and results of frozen section examination, total thyroidectomy plus bilateral CLND was performed in 174 (22.2%) patients, lobectomy plus ipsilateral CLND in 576 (73.4%) patients, and total thyroidectomy plus bilateral CLND and unilateral LLND in 35 (4.5%) patients. According to pathological examination of surgical specimens, LNM was found in 236 (30.1%) patients, among which 207 (26.4%) had only CLNM. LLNM was found in 29 (3.69%) patients. 7 (0.9%) patients had LLNM without CLNM (leap metastasis). Multifocality was found in 226 (28.8%) patients, among whom 86 (11.0%) had unilateral lesions and 140 (17.8%) had bilateral lesions.

### 3.2. Risk Factors for LNM in PTMC

**Figure 1:**
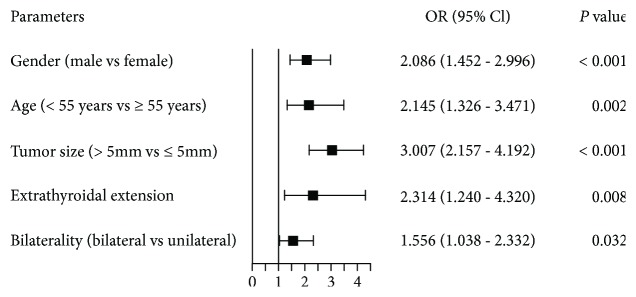
Multivariate analysis of clinicopathological factors predictive of lymph node metastasis (LNM).

We first analyzed the association between clinical parameters and LNM in PTMC. The American Joint Committee on Cancer has recently updated the 8^th^ edition of TNM staging for differentiated thyroid cancer, in which the age cutoff was raised from 45 to 55 years [9]. Thus, we accordingly divide patients into two age groups by 55 years, rather than 45. Univariate analysis showed that LNM was significantly associated with gender, age, tumor size, extrathyroidal extension, multifocality, and bilaterality, but not with lymphocytic thyroiditis complication or tumor location (Table 2). For multivariate analysis, five variables were included in the logistic regression model. The results were shown in Figure 1. In our model, male gender (OR = 2.086, *P* < 0.001), age < 55 years (OR = 2.145, *P* = 0.002), tumor size > 5 mm (OR = 3.007, *P* < 0.001), bilateral lesions (OR = 1.556, *P* = 0.032), and extrathyroidal extension (OR = 2.314, *P* = 0.008) remain to be independent predictive factors for LNM in PTMC.

### 3.3. Risk Factors for CLNM and LLNM in PTMC

**Figure 2:**
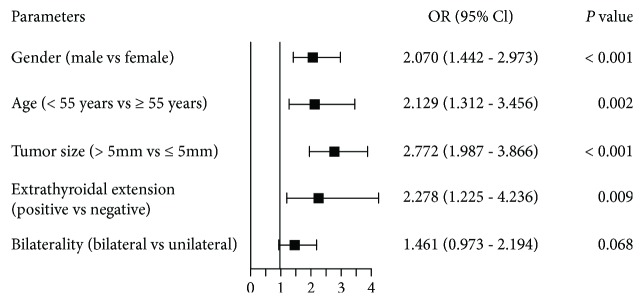
Multivariate analysis of clinicopathological factors predictive of central lymph node metastasis (CLNM).

The association between clinical parameters and CLNM/LLNM was further investigated. In univariate analysis, CLNM was significantly associated with gender, age, tumor size, extrathyroidal extension, multifocality, and bilaterality (Table 3). Again, five variables were included in multivariate analysis, which showed male gender (OR = 2.070, *P* < 0.001), age < 55 years (OR = 2.129, *P* = 0.002), tumor size > 5 mm (OR = 2.772, *P* < 0.001), and extrathyroidal extension (OR = 2.278, *P* = 0.009), but not bilaterality (*P* = 0.068), were independent risk factors for CLNM (Figure 2).

**Figure 3:**
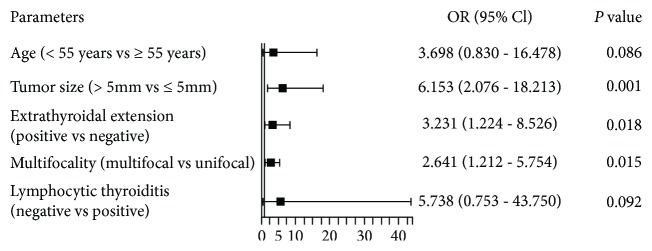
Multivariate analysis of clinicopathological factors predictive of lateral lymph node metastasis (LLNM).

LLNM was significantly associated with tumor size, extrathyroidal extension, multifocality, and bilaterality (Table 3). Unlike CLNM or LNM in general, the association between LLNM and gender or age was not significant. Besides, complication with lymphocytic thyroiditis had a marginal significant association with lower risk of LLNM (*P* = 0.070). Multivariate analysis included five variables, among which tumor size (OR = 6.153, *P* = 0.001), extrathyroidal extension (OR = 3.231, *P* = 0.018), and multifocality (OR = 2.641, *P* = 0.015) were independent risk factors for LLNM (Figure 3).

## 4. Discussion

As the ultrasound and imaging technique develop, preoperative identification or suspicion of PTMC has been increasingly common. There are controversies concerning surgical treatment for PTMC, especially in regional lymph node dissection. Risk stratification may indicate that different groups of patients require different treatments.

In our cohort, LNM was evident in 236 (30.2%) patients, similar to previous data of the LNM rate in nonincidental PTMC (30.0%) [10]. Skip metastasis (LLNM without CLNM) was reported to be more common in PTMC patients [11]. In this study, 7 (24% of patients with LLNM) patients had skip metastasis; the prevalence of which was similar to that in previous reports [12, 13].

In agreement with results of several previous studies, male gender was found to be an independent predictor for LNM and CLNM [14–16] in PTMC, but controversial for LLNM [12, 17–20]. A recent meta-analysis revealed that the male gender was significantly associated with LLNM in all sizes of PTC, with a pooled OR of 1.72 [21].

Numerous studies indicated that younger age (<45 years) is associated with higher risk of LNM [14, 15, 17]. According to the AJCC 8^th^ edition of TNM staging for differentiated thyroid cancer, all patients < 55 years have stage I disease if they do not have distant metastasis; otherwise, their disease is stage II [9]. Thus, 55 years is now considered as an important age cutoff with respect to the risk of mortality. We set 55 instead of 45 years as the age cutoff to keep consistency with the novel risk stratification system. Interestingly, under this cutoff, younger age is still an independent risk factor for LNM and CLNM. Ito et al. reported that young age was an independent predictor of PTMC progression under observation, including novel LNM [22]. Based on the longer survival time as well as higher risk of LNM, central lymph node dissection is particularly necessary for younger patients. Older PTMC patients without other risk factors may be the best candidates for observation. For LLNM, the role of age in predicting LLNM risk is debatable [12, 17–19].

The primary tumor size has been addressed in most similar studies, mostly with a cutoff of 5 mm. Other cutoff values of tumor size such as 7 mm [23] or 5.75 mm [15] have also been used. Larger size (>5 mm) of PTMC was significantly associated with higher frequency of CLNM [14]. Consistently, a tumor size of >5 mm was an independent risk factor for both CLNM and LLNM in our analysis.

In this study, extrathyroidal extension (ETE) was a significant predictor for all types of cervical LNM of PTMC, consistent with previous studies [14, 16, 20].

Multifocality was found previously to be associated with LNM in the central or lateral compartment [14]. Wang et al. demonstrated in a cohort of more than 2000 patients that among patients who have multifocal PTC, those with bilateral disease have a more advanced stage and shorter DFS, due to their higher incidence of lymph node metastasis [24]. Consistently, in our results concerning PTMC, bilaterality was correlated with higher risk of LNM and CLNM and it was a better predictor than multifocality in these analyses. However, for LLNM, bilaterality was less significant than multifocality in risk prediction. Because these two features were partially overlapped, we selected the stronger predictor between multifocality and bilaterality in the multivariate regression model for each analysis.

In PTMC, the association between chronic lymphocytic thyroiditis (CLT) coexistence and LNM risk is not yet clear. Zhang et al. found no association between CLT and cervical LNM in all sizes of PTC [25]. Jara et al. reported that in all sizes of PTC, the presence of CLT was associated with decreased risks for CLNM [26]. In our results, lymphocytic thyroiditis tended to be associated with lower LLNM frequency (0.8% versus 4.2%), though not statistically significant.

A few studies have analyzed the predictive value of several ultrasound characteristics of the primary nodule for LNM. In the study of Gui et al. [15], LNM was not correlated with composition, echogenicity, calcification, or spiculated margin. In multivariate models of Luo et al. [17], PTMC with nonuniform echoic distribution was prone to have CLNM and PTMC with ultrasound mix echo was prone to have LLNM. In our study, preoperative US characteristics were not included due to the lack of standardization of diagnostic techniques and report formats.

The major limitation of the current study is that it is a retrospective, single-institution study. Other limitations include the following: we did not distinguish the pathological subtypes of PTMC, preoperative ultrasound characteristics were not included, and LLND was only performed in patients who were diagnosed or suspected to have lateral lymph node metastasis; there might be bias in the analysis of lateral lymph node metastasis.

## 5. Conclusion

In PTMC, male gender, age < 55 years, tumor size > 5 mm, bilateral lesions and extrathyroidal extension are independent risk factors for LNM in general and CLNM. Prophylactic central lymph node dissection needs to be considered for clinically node-negative PTMC patients presenting with these risk factors. Tumor size > 5 mm, multifocal lesions and extrathyroidal extension are independent risk factors for LLNM. Clinical management for PTMC should be individualized based on risk stratification.

## Figures and Tables

**Table 1:** Patient characteristics.

Parameters	Patient number *n* (%)
Gender	
Male	189 (24.1%)
Female	596 (75.9%)
Age (year)	45.5 ± 10.0
Tumor size (mm)	5.73 ± 2.39
Multifocality	
Solitary lesion	559 (71.2%)
Multifocal lesions	226 (28.8%)
Unilateral	86 (11.0%)
Bilateral	140 (17.8%)
Surgery procedure	
Lobectomy plus ipsilateral CLND	576 (73.4%)
Total thyroidectomy plus bilateral CLND	174 (22.2%)
Total thyroidectomy plus bilateral CLND and unilateral LLND	35 (4.5%)
Open surgery	624 (79.5%)
Laparoscopic surgery	161 (20.5%)
LNM	
LNM	236 (30.1%)
CLNM only	207 (26.4%)
LLNM only	7 (0.9%)
CLNM and LLNM	22 (2.8%)

CLND: central lymph node dissection; LLND: lateral lymph node dissection; LNM: lymph node metastasis; CLNM: central lymph node metastasis; LLNM: lateral lymph node metastasis.

**Table 2:** Univariate analysis of clinicopathological factors predictive of lymph node metastasis (LNM).

Parameters	Total	LNM (-)	LNM (+)	*P*
Gender				
Male	189	109 (57.7%)	80 (42.3%)	<0.001
Female	596	440 (73.8%)	156 (26.2%)	
Age (years)				
<55	653	444 (68.0%)	209 (32.0%)	0.008
≥55	132	105 (79.5%)	27 (20.5%)	
Tumor size				
≤5 mm	417	338 (81.1%)	79 (18.9%)	<0.001
>5 mm	368	211 (57.3%)	157 (42.7%)	
Extrathyroidal extension				
No	736	528 (71.7%)	208 (28.3%)	<0.001
Yes	49	21 (42.9%)	28 (57.1%)	
Multifocality				
Unifocal	559	406 (72.6%)	153 (27.4%)	0.010
Multifocal	226	143 (63.3%)	83 (36.7%)	
Bilaterality				
Unilateral	645	464 (71.9%)	181 (28.1%)	0.009
Bilateral	140	85 (60.7%)	55 (39.3%)	
Complicated with lymphocytic thyroiditis				
No	664	462 (69.6%)	202 (30.4%)	0.608
Yes	121	87 (71.9%)	34 (28.1%)	

LNM: lymph node metastasis.

**Table 3:** Univariate analysis of clinicopathological factors predictive of central/lateral lymph node metastasis (CLNM/LLNM).

Parameters	Total	CLNM (-)	CLNM (+)	*P*	LLNM (-)	LLNM (+)	*P*
Gender							
Male	189	111 (58.7%)	78 (41.3%)	<0.001	180 (95.2%)	9 (4.8%)	0.372
Female	596	445 (74.7%)	151 (25.3%)		576 (96.6%)	20 (3.4%)	
Age							
<55	653	450 (68.9%)	203 (31.1%)	0.009	626 (95.9%)	27 (4.1%)	0.205
≥55	132	106 (80.3%)	26 (19.7%)		130 (98.5%)	2 (1.5%)	
Tumor size							
≤5 mm	417	338 (81.1%)	79 (18.9%)	<0.001	413 (99.0%)	4 (1.0%)	<0.001
>5 mm	368	218 (59.2%)	150 (40.8%)		343 (93.2%)	25 (6.8%)	
Extrathyroidal extension							
No	736	534 (72.6%)	202 (27.4%)	<0.001	714 (97.0%)	22 (3.0%)	<0.001
Yes	49	22 (44.9%)	27 (55.1%)		42 (85.7%)	7 (14.3%)	
Multifocality							
Unifocal	559	409 (73.2%)	150 (26.8%)	0.023	546 (97.7%)	13 (2.3%)	0.001
Multifocal	226	147 (65.0%)	79 (35.0%)		210 (92.9%)	16 (7.1%)	
Bilaterality							
Unilateral	645	468 (72.6%)	177 (27.4%)	0.022	627 (97.2%)	18 (2.8%)	0.004
Bilateral	140	88 (62.9%)	52 (37.1%)		129 (92.1%)	11 (7.9%)	
Complicated with lymphocytic thyroiditis							
No	664	469 (70.6%)	195 (29.4%)	0.778	636 (95.8%)	28 (4.2%)	0.070
Yes	121	87 (71.9%)	34 (28.1%)		120 (99.2%)	1 (0.8%)	

CLNM: central lymph node metastasis; LLNM: lateral lymph node metastasis.

## Data Availability

The raw clinical data used to support the findings of this study are available from the corresponding author upon request.

## References

[1] Hay I. D., Hutchinson M. E., Gonzalez-Losada T. (2008). Papillary thyroid microcarcinoma: a study of 900 cases observed in a 60-year period. *Surgery*.

[2] Lundgren C. I., Hall P., Dickman P. W., Zedenius J. (2006). Clinically significant prognostic factors for differentiated thyroid carcinoma: a population-based, nested case-control study. *Cancer*.

[3] Liu F. H., Kuo S. F., Hsueh C., Chao T. C., Lin J. D. (2015). Postoperative recurrence of papillary thyroid carcinoma with lymph node metastasis. *Journal of Surgical Oncology*.

[4] Mercante G., Frasoldati A., Pedroni C. (2009). Prognostic factors affecting neck lymph node recurrence and distant metastasis in papillary microcarcinoma of the thyroid: results of a study in 445 patients. *Thyroid*.

[5] Haugen B. R., Alexander E. K., Bible K. C. (2016). 2015 American Thyroid Association management guidelines for adult patients with thyroid nodules and differentiated thyroid cancer: the American Thyroid Association Guidelines Task Force on thyroid nodules and differentiated thyroid cancer. *Thyroid*.

[6] So Y. K., Seo M. Y., Son Y. I. (2012). Prophylactic central lymph node dissection for clinically node-negative papillary thyroid microcarcinoma: influence on serum thyroglobulin level, recurrence rate, and postoperative complications. *Surgery*.

[7] Caliskan M., Park J. H., Jeong J. S. (2012). Role of prophylactic ipsilateral central compartment lymph node dissection in papillary thyroid microcarcinoma. *Endocrine Journal*.

[8] Moon W. J., Jung S. L., Lee J. H. (2008). Benign and malignant thyroid nodules: US differentiation--multicenter retrospective study. *Radiology*.

[9] Perrier N. D., Brierley J. D., Tuttle R. M. (2018). Differentiated and anaplastic thyroid carcinoma: major changes in the American Joint Committee on Cancer eighth edition cancer staging manual. *CA: A Cancer Journal for Clinicians*.

[10] Mehanna H., al-maqbili T., Carter B. (2014). Differences in the recurrence and mortality outcomes rates of incidental and nonincidental papillary thyroid microcarcinoma: a systematic review and meta-analysis of 21 329 person-years of follow-up. *The Journal of Clinical Endocrinology & Metabolism*.

[11] Nie X., Tan Z., Ge M. (2017). Skip metastasis in papillary thyroid carcinoma is difficult to predict in clinical practice. *BMC Cancer*.

[12] Zhang L., Wei W. J., Ji Q. H. (2012). Risk factors for neck nodal metastasis in papillary thyroid microcarcinoma: a study of 1066 patients. *The Journal of Clinical Endocrinology & Metabolism*.

[13] Park J. H., Lee Y. S., Kim B. W., Chang H. S., Park C. S. (2012). Skip lateral neck node metastases in papillary thyroid carcinoma. *World Journal of Surgery*.

[14] Qu N., Zhang L., Ji Q. H. (2015). Risk factors for central compartment lymph node metastasis in papillary thyroid microcarcinoma: a meta-analysis. *World Journal of Surgery*.

[15] Gui C. Y., Qiu S. L., Peng Z. H., Wang M. (2018). Clinical and pathologic predictors of central lymph node metastasis in papillary thyroid microcarcinoma: a retrospective cohort study. *Journal of Endocrinological Investigation*.

[16] Wang Y., Guan Q., Xiang J. (2018). Nomogram for predicting central lymph node metastasis in papillary thyroid microcarcinoma: a retrospective cohort study of 8668 patients. *International Journal of Surgery*.

[17] Luo Y., Zhao Y., Chen K. (2018). Clinical analysis of cervical lymph node metastasis risk factors in patients with papillary thyroid microcarcinoma. *Journal of Endocrinological Investigation*.

[18] Kwak J. Y., Kim E. K., Kim M. J. (2009). Papillary microcarcinoma of the thyroid: predicting factors of lateral neck node metastasis. *Annals of Surgical Oncology*.

[19] Jeon M. J., Chung M. S., Kwon H. (2017). Features of papillary thyroid microcarcinoma associated with lateral cervical lymph node metastasis. *Clinical Endocrinology*.

[20] Kim Y. S. (2012). Patterns and predictive factors of lateral lymph node metastasis in papillary thyroid microcarcinoma. *Otolaryngology-Head and Neck Surgery*.

[21] So Y. K., Kim M. J., Kim S., Son Y. I. (2018). Lateral lymph node metastasis in papillary thyroid carcinoma: a systematic review and meta-analysis for prevalence, risk factors, and location. *International Journal of Surgery*.

[22] Ito Y., Miyauchi A., Kihara M., Higashiyama T., Kobayashi K., Miya A. (2014). Patient age is significantly related to the progression of papillary microcarcinoma of the thyroid under observation. *Thyroid*.

[23] Lee K. J., Cho Y. J., Kim S. J. (2011). Analysis of the clinicopathologic features of papillary thyroid microcarcinoma based on 7-mm tumor size. *World Journal of Surgery*.

[24] Wang W., Su X., He K. (2016). Comparison of the clinicopathologic features and prognosis of bilateral versus unilateral multifocal papillary thyroid cancer: an updated study with more than 2000 consecutive patients. *Cancer*.

[25] Zhang Y., Ma X. P., Deng F. S. (2014). The effect of chronic lymphocytic thyroiditis on patients with thyroid cancer. *World Journal of Surgical Oncology*.

[26] Jara S. M., Carson K. A., Pai S. I. (2013). The relationship between chronic lymphocytic thyroiditis and central neck lymph node metastasis in North American patients with papillary thyroid carcinoma. *Surgery*.

